# Effect of No-ozone Cold Plasma on Regeneration after Crushed Mental Nerve Injury in rats

**DOI:** 10.7150/ijms.77484

**Published:** 2022-09-25

**Authors:** Si-Yeon Park, M Shriya Jaiswal, Yoon-Seo Jang, Jeong-Hae Choi, Gyoo-Cheon Kim, Jin-Woo Hong, Dae-Seok Hwang

**Affiliations:** 1 Department of Oral and Maxillofacial Surgery, Dental and Life Science Institute, Dental School, Pusan National University, Busan, South Korea.; 2 Department of Research and Development, FEAGLE Corporations, 70‑6, Jeungsan‑ro, Mulgeum‑eup, Yangsan‑si, Gyeongsangnam‑do 50614, South Korea.; 3 Department of Translational Dental Science, School of Dentistry, Pusan National University, Beomeo-ri, Mulgeum-eup, Yangsan-si, Gyeongsangnam-do, 50612, South Korea.; 4 Department of Internal Medicine, School of Korean Medicine, Yangsan Campus of Pusan National University, Yangsan-si, Gyeongsangnam-do, 50612, South Korea.; 5 Dental Research Institute, Pusan National University Dental Hospital, Yangsan, South Korea.

**Keywords:** No-ozone Cold Plasma (NCP), Peripheral nerve injury, mental Nerve Crush Injury, Skeletal Muscle Healing, Macrophage, Myelin Sheath, Neuronal Axon.

## Abstract

**Background:** This experimental research aimed to determine whether No-ozone Cold Plasma (NCP) has regenerative effect on crushed injured sensory nerves in a rat model (Wistar A) and to evaluate whether NCP can be used as an alternative treatment method for sensory nerve injury in the oral-maxillofacial region.

**Methods:** A total of 10 Wistar A rats were used for this experiment. They were divided into three groups according to whether the mental nerve of the left mandible was injured and NCP was applied or not: group 1 (n=3) (non-mental nerve damage, non-MD) - the left mental nerve was exposed and non-damaged; group 2 (n=3) (mental nerve damage, MD) - the left mental nerve was exposed and damaged, NCP was not applied; and group 3 (n=4) (mental nerve damage and NCP, MD-NCP) - the left mental nerve was exposed and damaged, NCP was applied with regular intervals (three times a week).

**Results:** For the behavior analysis, von Frey test was used. Furthermore, the nerve tissues were examined with hematoxylin and eosin (H&E) staining, and the extent of neurorecovery was evaluated with the immunofluorescence staining of certain markers. The behavioral analysis showed that the function recovery sensory nerve was faster in group 3 (MD-NCP). In the histomorphologic and immunofluorescence analyses, the expression of the factors involved in neurorecovery was much higher in group 3 than in group 2 (MD).

**Conclusions:** The expeditious recovery of sensory nerve function as well as the higher expression of the factors indicating nerve function recovery in the NCP-treated group suggest that NCP has a positive effect on regeneration after sensory nerve crushing injury. Therefore, in the case of sensory impairment of the oral-maxillofacial region, no-ozone cold plasma can be applied for therapeutic effect.

## Introduction

Dental implants have become commonplace for oral rehabilitation. As the use of dental implants increases, the frequency of side effects from them also increases. The most representative of these side effects is nerve damage due to implant placement 1, 2.

In the oral and maxillofacial region, the trigeminal nerve dominates the senses. The mental nerve is the most injured terminal branch of trigeminal nerves during oral and maxillofacial treatment 3, 4. Nerve damage in the oral and maxillofacial area interferes with daily life, which adversely affects the patient's psychosocial state and causes his or her quality of life to deteriorate 5. There have been reports of permanent neurological damage from 0 to 36% causing lip paresthesia after implant placement 6-8, but recent studies have reported that 2.95% of patients have transient neurological damage and 1.7% have permanent neurological damage 9, 10.

The over prepared implant hole allows the implant to enter the nerve canal or directly damage the nerve bundle 11. This process usually results in nerve compression or transection damage. When nerve damage occurs due to implant placement, clinicians must decide whether to do nerve anastomosis, treat it with pharmacologic agents only, or remove the implant immediately.

Surgical methods like nerve anastomosis or nerve graft are the gold standard for the treatment of nerve injury, but because the micro surgeon can perform microsurgery only if the equipment is available, general clinics cannot easily perform it 12-15. The postoperative repair and sensory nerve function recovery rates range from 50 to 60%, and the methods have been reported to be effective in some patients 16-20.Vitamin B, ATP, steroid, and alpha lipoic acid (ALA) are used as pharmacologic agents, but these have not yet been proven to be effective 21.

The use of plasma is one of the newly introduced alternative methods of treatment for nerve injury. Plasma is the fourth state of matter and has high energy. After its introduction in the 1850s, Plasma, a biomedicine, was introduced in the 21^st^ century 22, 23.

Earlier, argon gas was used to reduce the amount of ozone and NO compounds generation from nozone (no-ozone) cold plasma device. However, lately Feagle Co. Ltd. (Yangsan, South Korea) has developed a NCP device, by which non-thermal plasma can be used with several applications. It has got distinctive features. It not only generate very limited amount of ozone but also maintain the optimum temperature of 35C or lower. This is the crucial feature in order to use the NCP in the study as the ozone and NO compounds can harm the respiratory system.

NCP contains reactive oxygen species, high-energy electrons, and ions. As such, it is known to modulate a number of biological responses, including inflammatory response 24, 25. NCP is known to be highly applicable to cancer treatment and skin anti-aging, sterilization, teeth bleaching and wound healing promotion 26-32.

In previous studies, after inflicting crush or cut injury to the sciatic nerve of rats, NCP was applied to investigate the therapeutic effect 33. These studies revealed that the function recovery and neuronal regeneration greatly improved after plasma application.

To quantify and measure the recovery of the function of the motor nerves, such as the sciatic nerve, the static sciatic index (SSI) can be used. It is difficult to quantify sensory nerve function recovery in animal experiments compared to motor nerve function recovery because animals cannot communicate their sensory experiences 34.

Thus, to test animals, sensory nerve function recovery, electrical, thermal, physical, or chemical stimuli can be used 35. For mechanical testing, the von Frey test is a representative method that can be used for both rats and mice 36. It makes use of the von Frey filament, a plastic filament with blunt ends that can apply 0.008-300 g pressure. Paw withdrawal, which occurs when pressure is applied to the experimental animals by the von Frey filament, indicates sensory nerve function recovery or pain. In the von Frey test in this study, however, pressure was applied to the facial part of the experimental animals, and head withdrawal was used to measure their sensory nerve function recovery.

The purpose of this study was to investigate the effects of NCP on nerve injury caused by crushing injury to the mental nerves of rats. This was the third study that evaluated NCP's effects on peripheral nerve regeneration, and the first using only a sensory nerve injury model.

## Materials & Methods

### Plasma generating device

**Figure 1:**
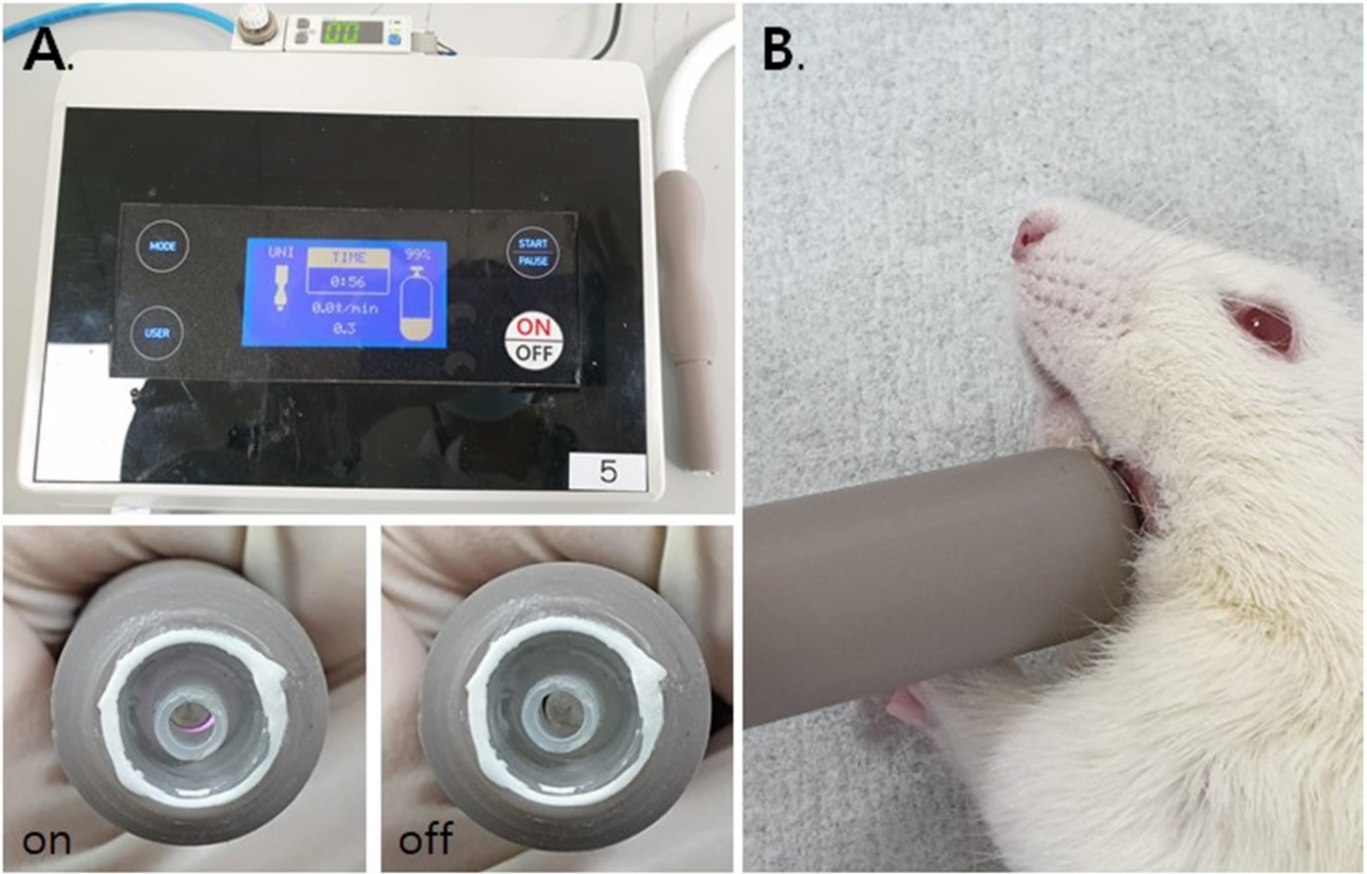
The NCP-generating device (A) and the NCP treating methods on rat (B).

For this study, a dielectric-barrier-discharge-(DBD)-type NCP device developed by FEAGLE Corporation (Yangsan-si, South Korea) was adopted. Argon gas was used as a buffer gas, and 2.0 slm (standard liter per minute) of it was blown into the plasma source. NCP was formed by applying a high voltage (3 kV) to the plasma source. Plasma glow was formed within the electrodes, but it did not extend to the end of the electrodes. The temperature of the NCP flow at 1 cm from the electrode end was maintained under 35°C for 10 minutes, and no UVs were detected at this condition. Along with that, the value of ozone in this device was found to be 0.006ppm which seems to be much less than the level of ozone recommended by Food and Drug Administration (FDA) that is 0.05ppm.The distance of the skin from the electrodes was kept at 1 cm [Figure 1].^33^, ^37^-^39^

### Animal experiments

**Figure 2:**
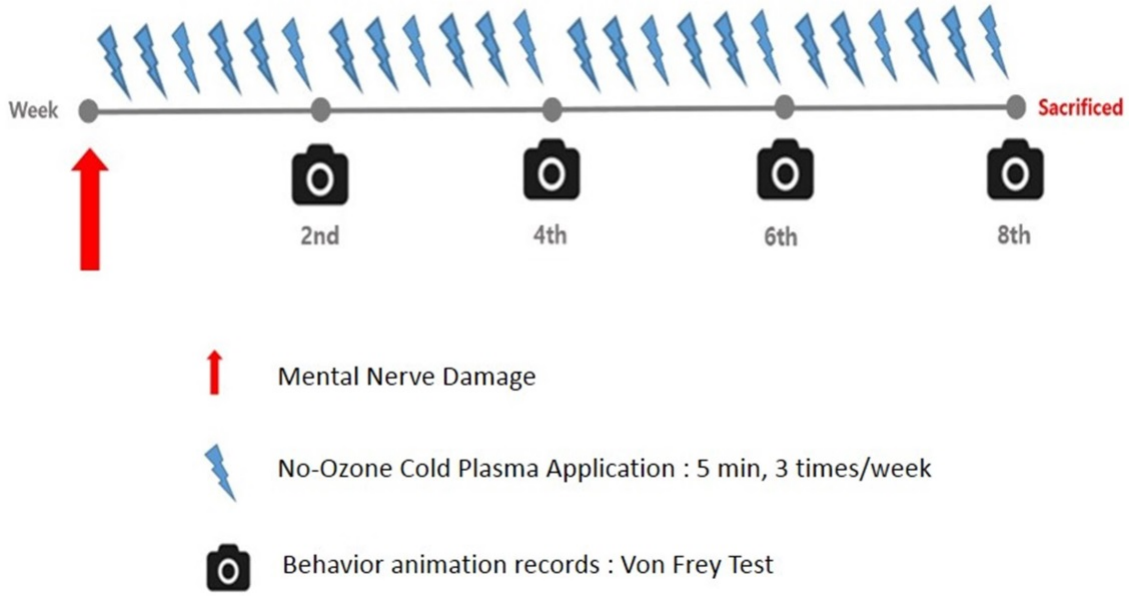
Time line: NCP application, nerve damage and behavior animation records.

A total of 10 male Wistar A rats 6 weeks of age and weighing between 200 and 225 grams were used. The nerves of the rats were so close to their skins that they did not require re-incision to apply NCP. Rats' nerves are very similar to the human mental nerve in this regard, so the use of rats is very suitable for the experiment. All the experiments were conducted in accordance with the ethical guidelines of Pusan National University Institutional Animal Care and Use Committee (PNUIACUC, South Korea) and the regulations set out by International Association for the Study of Pain (IASP) in Animals (PNU-2019-2276). It is also complied with the ARRIVE guidelines. The experiment was conducted on three groups. Group 1 (n=3) was the control group, which underwent the same operation as that undergone by another group, but without any nerve injury. Groups 2 (n=3) and 3 (n=4) underwent the same surgical procedure, but only group 3 received NCP to the skin overlying the injured mental nerve 3 times a week for 8 weeks [Table 1] [Figure 2].

After the completion of the experiment, animal euthanasia was performed using CO2 gas chamber. The cage was kept as it was in CO2 gas chamber and the flow of the gas was maintained for about 1-3 minutes until the death was observed.

### Surgical procedure

To anesthetize the animals, Intraperitoneal (IP) injection of a cocktail of 100 mg/kg Ketamine (Yuhan, Seoul, South Korea) and 10 mg/kg Xylazine (Rompun, BAYER KOREA Ltd., Seoul, South Korea) was used. A 0.3mL solution containing 1:100000 epinephrine and 2% lidocaine HCl was used for the local anesthesia, and cefazolin (50 mg/kg) was injected preoperatively subcutaneously, as a prophylactic antibiotic. After complete anesthesia was achieved, the rats were fixed with pins in a lateral position on the disinfected surgical plate. The surgical area of each rat's left mandible was shaved using an electric clipper, and was scrubbed with povidone iodine solution for disinfection. An about 1-cm-long skin incision was made at an angle from the left mandible, and the subcutaneous tissue was carefully dissected, with the mental nerve exposed. In group 1, the skin layer was sutured with 6-0 ETHILON synthetic non-absorbable monofilament suture in a continuous pattern, without any nerve injury. In groups 2 and 3, the mental nerve was crushed with a hemostat 5 mm proximal to the mental foramen for 30 seconds, and then skin suture was done with the same pattern as in group 1. Group 1 and Group 2 were not treated with NCP. In group 3, NCP was applied to the skin over the damaged mental nerve.

**Figure 3:**
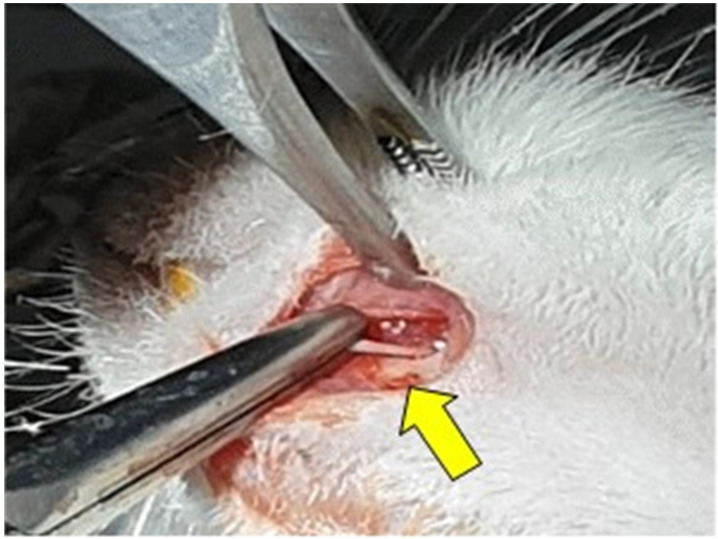
Nerve injury: crushing with hemostat (yellow arrow).

After NCP treatment, rats were anesthetized, and the NCP was irradiated for 5 minutes at the distance of 0.5 cm from the skin. [Figure 3].

### Behavior analysis

Behavioral analysis was conducted in the morning for 8 weeks, once every 2 weeks from 2 weeks after the surgery. Each rat's body was fixed in a cage made of a transparent acrylic plate (HEAD OUT RESTRAINER, Jeung Do Bio & Plant Co., Ltd., Seoul, South Korea), but even when the body was locked in the box, the rat's head was not fixed and could move freely. Behavior analysis was performed with a tablet PC (iPad Pro, Apple, California, USA) installed 20 cm away from the front of the box, and the behavioral experiment was recorded in a video.

**Figure 4:**
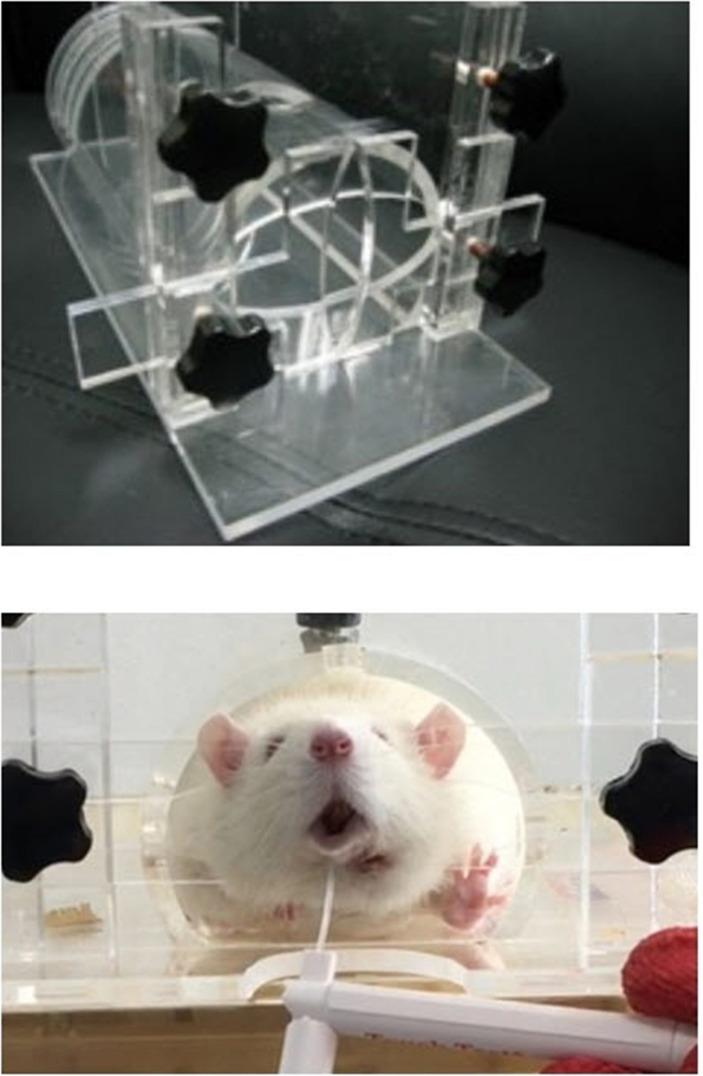
Head out restrainer (Jeung Do Bio & Plant Co., LTD. Seoul, South Korea) and the Withdrawal test using von Frey filament.

The withdrawal test with von Frey filament (Stoelting Co., Wood Dale, IL, USA) was used for behavioral analysis. Withdrawal response was defined as the case where there is a movement opposite the direction of filament application. A 300g filament force was applied to both the left and right sides of each rat's lower lip. The withdrawal response rate difference between the experiment and opposite sites was recorded through video playback [Figure 4].

### Histomorphologic analysis

One day after the final NCP treatment, all the experimental rats were sacrificed, and the nerve tissues of the surgical areas were isolated. All the tissue samples were fixed with 4% paraformaldehyde for 24 hr., and then embedded in paraffin. The tissue sections (5 μm thick) were subjected to hematoxylin and eosin (H&E) staining, and the morphology of the nerve tissues was then visualized by taking pictures of them using an iCM 9.0 digital camera system (IMT I-solution Inc., NY, USA) coupled with light microscopy (CX31, Olympus, Tokyo, Japan).

### Immunofluorescence analysis

The 5-μm-thick tissue sections were treated with antibodies against CD68 and myelin basic protein (Santa-Cruz Biotechnology, Santa Cruz, CA, USA), neurofilament 200, S100, and tau (Abcam, Cambridge, MA, USA), and were incubated for 2 h at 37℃. After being washed 4 times with phosphate-buffered saline, the tissue sections were treated with anti-mouse Alexa Fluor-488 and anti-rabbit Alexa Fluor-594 (Thermo Fisher Scientific, Rockford, IL, USA) for 1 h at 37℃. After the washing, the nuclei of the cells within the tissues were counterstained with 4', 6-diamidino-2-phenylindole. The fluorescence from the tissues was observed, and images were captured using a Carl Zeiss LSM 780 confocal laser microscope.

### Statistical analysis

Each experiment was repeated three times with standard deviations indicated as error bars. Experimental and control groups were compared using Excel's paired t-test (Microsoft, Redmond, WA, USA). Statistical significance was determined to be p < 0.05.

## Results

### Animal Behavioral Analysis showed that NCP treatment accelerated the recovery rate of mental nerve damage

**Figure 5:**
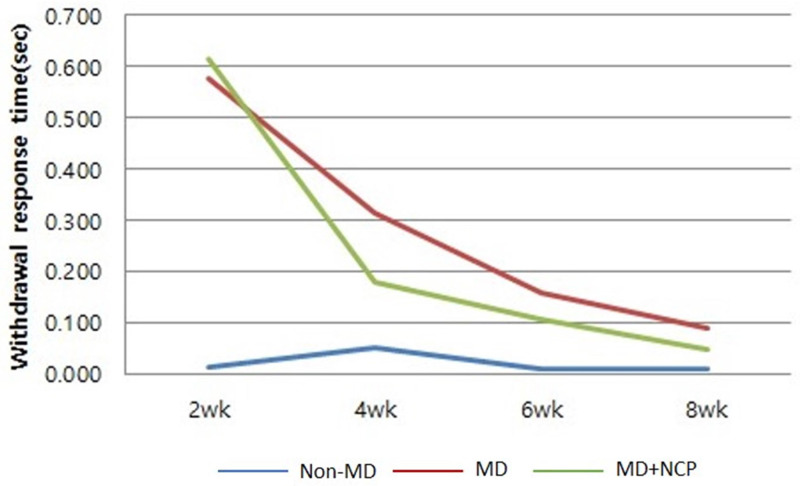
The effect of NCP on mental nerve crushing damage recovery: Withdrawal response time. At week 4, the withdrawal response time rate was significantly decreased in group 3 than in group 2.

The behavioral recovery of the mental nerve was evaluated to address sensory nerve function recovery. In group 1 (non-mental-nerve damage, non-MD), within 2 weeks, the withdrawal response time was already almost the same as that on the unaffected right side. In groups 2 (mental nerve damage, MD) and 3 (mental nerve damage-NCP, MD-NCP), there was no significant difference in withdrawal response time at week 2. At week 4, the withdrawal response time rate was significantly decreased in group 3 than in group 2. At week 8, the withdrawal response time rate was already on the same level as that on the unaffected right side in both groups 2 and 3, but the recovery was slightly greater in group 3 [Figure 5].

### NCP treatment accelerated the recovery of damaged areas of the mental nerve

**Figure 6:**
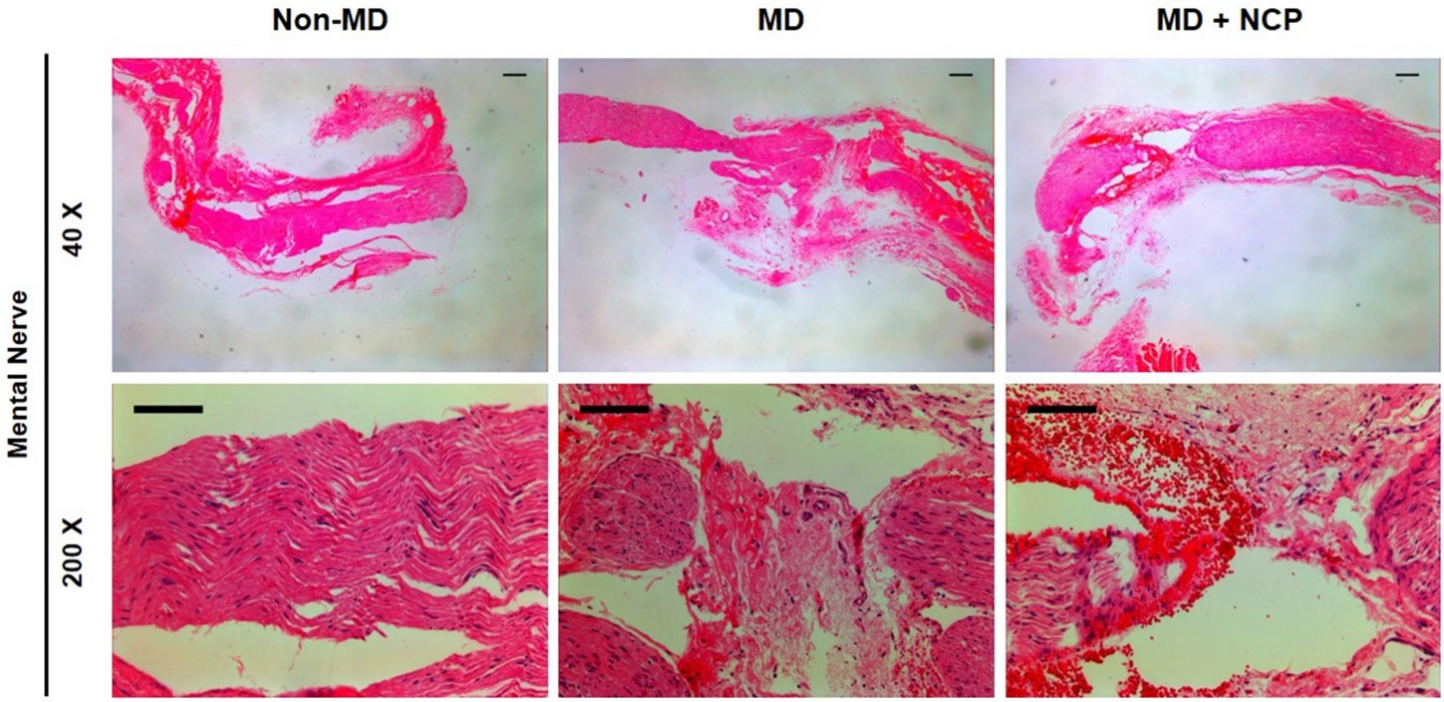
The effect of NCP on mental nerve crushing damage recovery: The number of cells is the highest in the NCP treated group (H&E stain). *Scale bars 100µm

The differences in neural tissue recovery among the three groups were also observed, as can be seen in [Figure 6]. The part of the mental nerve where crushing damage was applied with a hemostat was observed under a microscope. In H&E staining, the nuclei of the cells were stained in violet to identify the cells, and the eosinophilic parts were the axon and myelin sheath of the nerve fibers 40. There was a clear difference in the number of cell nuclei between groups 2 and 3. Obviously, there was a large number of cell nuclei in group 3, suggesting that the number of cells was higher.

### NCP restored the neuronal axon and myelin sheath and decreased the overexpression of CD68 positive macrophage

Immunofluorescence confirmed the markers for nerve regeneration, and compared the differences between the groups.

**Figure 7:**
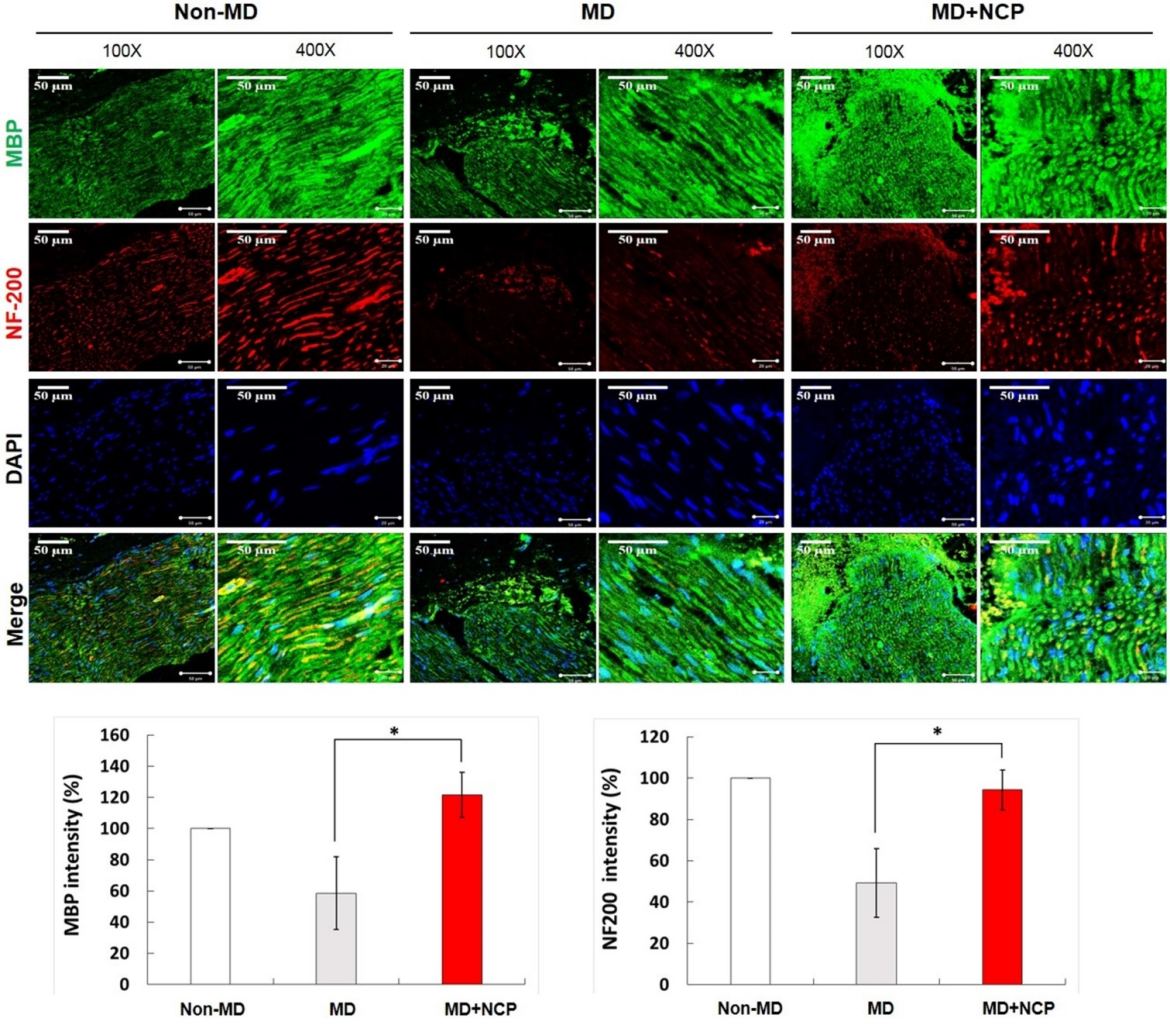
Immunofluorescence in neural tissue and MBP and NF-200 intensity (%) in the three groups. In the NCP treatment group, both MBP and DAPI stained more intensely. * MBP: Myelin Basic Protein, myelin sheath marker. * DAPI: 4', 6-diamidino-2-phenylindole. * Scale bars 50µm

Compared with MBP, the crushed part of MD, the MD+NCP group was broken, but the MD group seemed to have maintained the damaged part as it was. The plasma-treated group, however, had more intense MBP expression, and it was observed that myelin was regenerated. [Figure 7].

CD68 and MAP2 were the most expressed in group 2, and their expression level in group 3 appeared to be similar to that in group 1.

NF-200 was highly expressed in group 3, as in group 1. In group 2, however, it was hardly expressed, and its continuity was lost, whereas in group 3, the continuity was recovered to the same level as in group 1. DAPI was more expressed in both groups 2 and 3 than in group 1, and was most expressed in group 3.

As for GAP 43, it was more expressed in groups 2 and 3 than in group 1, and it was more expressed in group 3 than in group 2.

**Figure 8:**
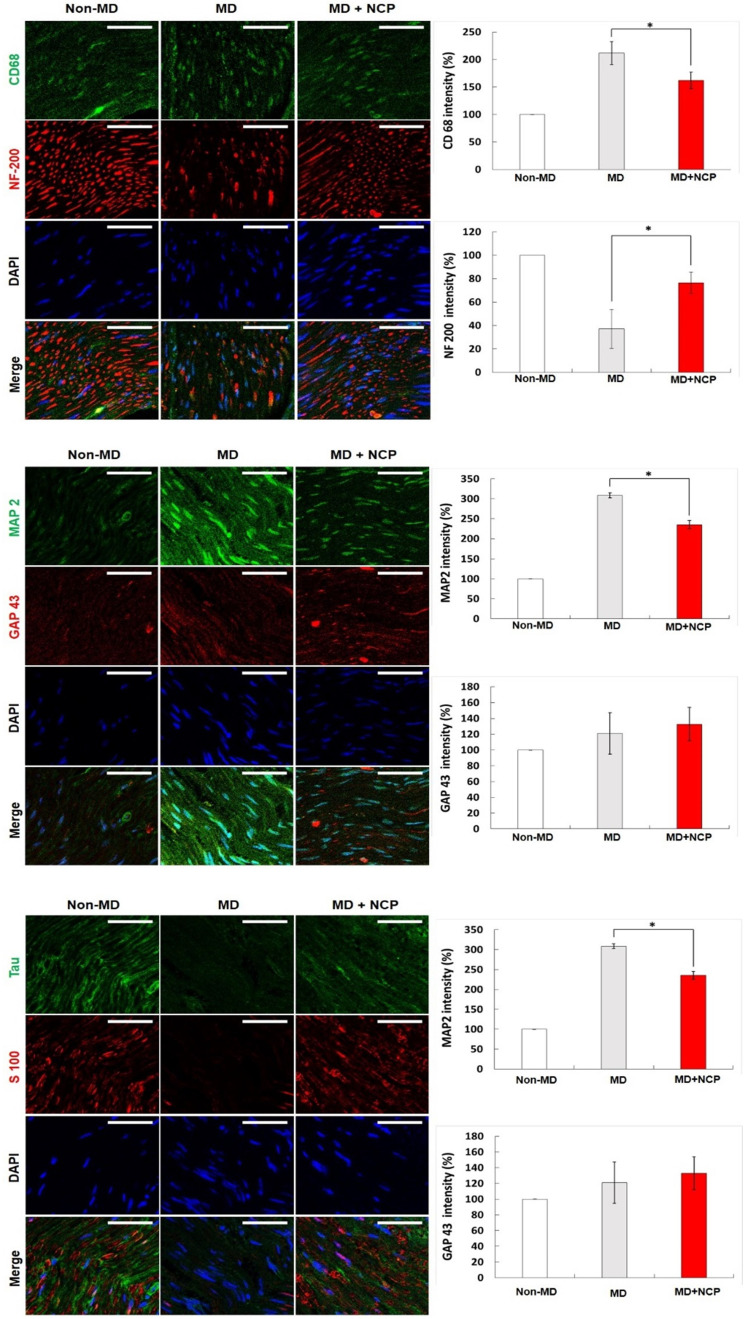
Immunofluorescence in neural tissue in three groups (x400): NF-200, CD68, MAP2 were weakly expressed and GAP43, S100 and tau were strongly expressed in the NTP treatment group. * NF-200: Neurofilament-heavy chain, mature nerve cell marker. * CD68: Macrophage marker. * MAP2: Microtubule-associated protein 2. * GAP43: growth-associated protein 43. * Scale bars 50µm.

S100, and tau were significantly less expressed in group 2 and were expressed at similar levels in groups 3 and 1. Overall, group 3, which was treated with NCP, showed immunofluorescent staining similar to that in group 1 compared to group 2 (without NCP) [Figure 8].

## Discussion

Plasma has been used biologically and medically for the last few decades. Low-pressure plasma, with the thermal effect of plasma, was initially mainly used, whereas No-ozone Cold Plasma (NCP) was developed and used in the early 1990s. The primary purpose of using plasma was material processing, such as rendering plastics or cloth more hydrophilic or hydrophobic. In the mid-1990s, however, the discovery of the bacterial inactivation or wound healing promotion effects of plasma led to the development of “plasma medicine” as the interest in the relationship between plasma and the biological cells increased 41, 42.

DBD, a type of NCP, is the source of reactive oxygen species (ROS) and reactive nitrogen species (RNS). OH, O, NO, H_2_O_2_, and O2^-^ are known to have biological implications. Through these active molecules (both radical and non-radical), NCP was found to break the “equilibrium” of the redox process, to stimulate cell signaling, and to affect the cells 41.

The effect of plasma has been proven in various medical fields, including dentistry, and its utilization is increasing due to the convenience of the DBD plasma equipment. In addition, the ability to control the tissue activity of plasma can be considered in the treatment of tissue damage, such as nerve damage that can occur in dentistry 43, 44

According to the results of a previous study of crush injury to the sciatic nerve, 3 weeks after the injury was incurred, the nerve function was fully recovered in the NCP-treated group whereas only 60% of the nerve function was recovered in the group whose injury was healed without NCP treatment 32. As NCP was applied to the rats' skins and muscles during the experiment, histological examination was performed considering the possibility of promoting wound healing. In the NCP-treated group, the diameter and density of the cells near the muscle injury increased. This was due to the NCP satellite cell proliferation promotion effect 45, which is effective for the recovery of the muscles around the nerves as well as of the motor nerves.

To assess the recovery of the motor function of the sciatic nerve in the rat model, SSI, which was introduced by Bervar in 2000, can be used 46. On the other hand, for analyzing sensory nerve function recovery, the von Frey test can be used. The von Frey filament is a plastic filament with blunt ends that can apply 0.008-300 g pressure. The filament is used to measure the amount of pressure needed to be applied to make an experimental rat withdraw its paw after its stimulation. To quantify, researchers can use the 50% threshold devised by Chaplan et al. 47.

In this study, the von Frey test was applied to the lower lip of the experimental rats. As the lower lips of rats, however, are covered by hair, unlike the paws, they are difficult to access, which limits the application of the von Frey filament thereto. In this experiment, the sensory nerve function recovery rate was determined by measuring the time to head evasion on the non-operated and surgical sides, with the gram of filament fixed.

Restrictions on the movement of rats into the restrainer can cause significant stress and can affect the experiment results. To prevent this, the restrainer was repeatedly adjusted for about 1 week before the experiment. In addition, the existing restrainer has a structure that suppresses the rat's neck in three directions (upper, left, and right), but the restrainer parts in the upper direction were removed in the experiment in this study. As such, it did not interfere with the breathing of the rats, but it inhibited the rats' movement and reduced the stress on them. In addition, the experiment was repeated to reduce the experiment errors and to improve the reproducibility.

There have been no previous studies that conducted the von Frey test on the faces of rats, which limits the quantification of sensory nerve function recovery. In this regard, further research on sensory measurement is needed. The results of the behavioral analysis in this study showed that after 4 weeks, the withdrawal response rate of group 3 increased even more significantly. This suggests that NCP can make a positive contribution in promoting the rate of sensory nerve function recovery in crushing-injured nerves.

In the H&E staining, much more nuclei were observed in the MD-NCP group. This may confirm that NCP stimulated cell division to restore the injured area.

MBP is produced in the Schwann cells, which contain myelin, essential for maintaining the structure and function of the peripheral nerves 48. Increased expression of MBP implies correction of the structural defects in damaged nerves. The expression of MBP increased in group 3 compared to group 2, which suggests that NCP promotes MBP expression and recovery of the function of injured mental nerves.

NF-200 is a marker found only in A-fiber, which is the myelinated nerve fiber distributed in the sensory nerves 49. In the MD group in this study, there was continuity of NF-200, but in group 2, there was none, and in group 3, it was gradually recovered. In addition, the expression of NF-200 was increased significantly more in group 3 than in group 2, which also supports the conclusion that NCP promotes repair of damaged nerves.

DAPI is a marker that can confirm the total number of cells by binding to double-stranded DNA. In this experiment, DAPI was expressed more in groups 2 and 3 than in group 1. This suggests that several cells actively proliferated to repair the nerve damage, and especially that the DAPI expression in group 3 was higher than that in group 2 because NCP promotes cell proliferation.

CD68 expressed by macrophage was more expressed in group 2. Overexpressed CD68 may also slow inflammation by making it become chronic. This suggests that the inflammatory response persisted in group 2, and that inflammation was suppressed and nerve regeneration progressed in group 3.

MAP2 is responsible for the production of somatodendritic cytoskeleton, and GAP43 is a factor that initially regulates axon growth and plasticity. Nerve regeneration is mainly performed by axon, and increased dendrite production prevents axonal growth 46. In group 3, which was treated with NCP, the dendrite production did not increase, suggesting that the environment for axon regeneration was well established.

The S100 and tau are essential for neuroskeleton and axon regeneration. Tau is one of the microtubule-associated proteins (MAPs) primarily present in the axons of normal neurons. It increases the stability of the microtubules *in vitro*. Although many studies on it have been reported, the actual role and importance of the tau cells have yet to be found and established. To date, research suggests that tau may be involved in axon development at the developmental stage of cells, and may regulate microtubule assembly and dynamics as well as the transport of axons in organelles like mitochondria. The significantly increased expression of tau in group 3 compared to group 2 in this study suggests that NCP promotes axon regeneration 50.

The aforementioned results are similar to those of previous studies that inflicted crushing and cut injury on the sciatic nerve. In the NCP-treated group, MBP, NF-200, GAP-43, S100B, and tau, which are involved in axonal regeneration, were increased, and CD68 and MAP2, which have negative effects on regeneration, were decreased 33.

The previous studies that analyzed the structural differences between the motor and sensory nerves showed that the motor neurons have more neurotrophic factors, such as insulin-like growth factor, basic fibroblast growth factor, and ciliary neurotrophic factor. It has also been reported that the motor neurons have larger Schwann cell basal lamina tubes than the sensory nerves. This resulted in more effective nerve regeneration when the motor nerve rather than the sensory nerve was grafted into the nerve gap. It can be inferred that structurally, the ability to regenerate damaged sensory nerves is slightly lower than the ability to regenerate damaged motor nerves. Therefore, the effect of NCP on sensory nerve function recovery after injury is more important. Such effect can be maximized by applying NCP to the injured sensory nerves that are slightly lacking in self-regeneration 51-57.

Both the histomorphologic and immunofluorescence analyses showed that NCP had a positive effect on promoting the recovery of the structure of the mental nerve with crushing injury. Also, in the behavioral analysis, NCP was found to have promoted the regeneration of the damaged sensory nerves. These results are in line with the results of the previous study. As this study was the first to have looked into the effect of NCP on the maxillofacial region, further study on the effect of NCP on the nerve regeneration of the maxillofacial region of other animal models is needed for the future application of NCP for nerve regeneration in the human maxillofacial region.

## Conclusion

The results of this study, which examined the nerve regeneration after applying NCP to the crushed/injured mental nerve of rats shows that the use of NCP can have a positive effect in the progression of nerve functional recovery and neuronal regeneration. This suggests the possibility of non-invasive treatment of sensory impairment in the oral and maxillofacial region, such as sensory impairment due to dental implant placement, etc.

## Figures and Tables

**Table 1:** Classification of Control and Experimental Groups.

	Exposure of Mental Nerve	Crush Injury of mental Nerve	NCP Applying	Number of Rats
**Group 1** **Non-mental Nerve Damage (Non-MD)**	**O**	**X**	**X**	**3**
**Group 2** **Mental Nerve Damage (MD)**	**O**	**O**	**X**	**3**
**Group 3** **Mental Nerve Damage with NCP (MD-NCP)**	**O**	**O**	**O**	**4**

**Non-MD:** non-mental nerve damage; **MD:** mental nerve exposed and damaged without NCP; **MD-NCP:** mental nerve exposed and damaged with NCP.

## Abbreviations

NCP: No-Ozone Cold Plasma
non-MD: non-Mental nerve damage
MD: Mental nerve damage
MD-NCP: Mental nerve damage + No-Ozone Cold Plasma
